# Nationwide trends and demographic projections of hospitalisations for vertebral fractures and postmenopausal osteoporosis in Germany: a 24-year registry-based study

**DOI:** 10.1007/s11657-026-01796-5

**Published:** 2026-10-30

**Authors:** Houmam Anees, Khaled Qadourah, Goran Georgievski, Christian Heiss, Thaqif El Khassawna

**Affiliations:** 1 Department of Trauma, Hand and Reconstructive Surgery, Faculty of Medicine, Justus-Liebig-University of Giessen, Giessen, Germany; 2 Experimental Trauma Surgery, Faculty of Medicine, Justus-Liebig-University of Giessen, Giessen, Germany; 3 Biruni University, Istanbul, Türkiye; 4 School of Pharmacy, The University of Jordan, Amman, Jordan

**Keywords:** Epidemiology, Hospitalisation, Demographic projections, Osteoporosis, Population ageing, Vertebral fractures

## Abstract

***Summary*:**

Hospitalisations for vertebral fractures increased in Germany over the past two decades. Although hospitalisation rates have recently stabilised or declined, demographic projections indicate that the absolute inpatient hospitalisation burden may remain substantial through 2050. These findings highlight the need for better osteoporosis prevention and long-term healthcare planning.

**Background:**

Vertebral fractures represent one of the most common manifestations of skeletal fragility and contribute substantially to morbidity, disability, and healthcare utilisation in ageing populations. However, nationwide long-term hospitalisation trends and the impact of demographic ageing on future inpatient burden remain insufficiently characterised. This study investigated inpatient hospitalisation trends for vertebral fractures and postmenopausal osteoporosis in Germany over a 24-year period and estimated the future inpatient burden through 2050.

**Methods:**

Nationwide inpatient data from the German Federal Health Monitoring (GBE) database were analysed between 2000 and 2023. Hospitalisations were identified using ICD-10-GM codes for thoracic vertebral fractures (S22.0), lumbar vertebral fractures (S32.0), and postmenopausal osteoporosis with osteoporotic fracture (M80.0). Absolute hospitalisations, age-standardised hospitalisation rates, sex-specific trends, in-hospital mortality, and length of hospital stay were evaluated. Temporal trends were assessed using joinpoint regression. Future hospitalisations were projected by applying age-specific hospitalisation rates observed during 2021–2023 to the official German population projections (Destatis main scenario G2L2W2).

**Results:**

Between 2000 and 2023, annual hospitalisations increased from 14,057 to 27,563 for thoracic vertebral fractures, from 27,683 to 45,280 for lumbar vertebral fractures, and from 2,617 to 11,800 for postmenopausal osteoporosis with osteoporotic fracture. In contrast, age-standardised hospitalisation rates increased until the mid-to-late 2010s before stabilising or declining across all three diagnoses. Joinpoint regression identified significant trend reversals for thoracic vertebral fractures (2016), lumbar vertebral fractures (2018), and postmenopausal osteoporosis with osteoporotic fracture (2015). Overall, lumbar vertebral fractures (AAPC − 0.65%, *p* = 0.01) and postmenopausal osteoporosis with osteoporotic fracture (AAPC − 1.17%, *p* < 0.001) demonstrated significant long-term declines, whereas thoracic vertebral fractures remained stable (AAPC − 0.44%, *p* = 0.10). Assuming stable age-specific hospitalisation rates, the demographic projection indicated an approximately 10% increase in annual inpatient hospitalisations for thoracic and lumbar vertebral fractures by 2050.

**Conclusions:**

Although age-standardised hospitalisation rates have stabilised or declined in recent years, demographic projections indicate that the inpatient hospitalisation burden associated with vertebral fractures may remain substantial in Germany. These findings underscore the need for timely osteoporosis diagnosis, systematic secondary fracture prevention, and long-term healthcare planning for an ageing population.

## Introduction

Osteoporosis is a systemic skeletal disease characterised by reduced bone mass and deterioration of bone microarchitecture, leading to increased bone fragility and susceptibility to fragility fractures [1]. It is among the most prevalent chronic musculoskeletal disorders worldwide and has become a major public health challenge owing to increasing life expectancy and population ageing [2]. In Germany, osteoporotic fractures are associated with considerable lifetime healthcare costs, with hospital treatment and long-term care representing the major contributors to the overall economic burden [3, 4]. Beyond reduced bone mineral density, osteoporosis is associated with substantial disability, excess mortality, impaired quality of life, and increasing healthcare resource utilisation following fragility fractures [2, 5].

Fragility fractures of the hip, spine, proximal humerus, and distal radius are commonly classified as major osteoporotic fractures [6, 7]. Among these, osteoporotic vertebral fractures (OVFs) are among the most common types of osteoporotic fracture, with approximately 1.5 million new cases occurring annually worldwide [6]. Clinically symptomatic vertebral fractures may be associated with persistent back pain, progressive kyphotic deformity, impaired physical function, reduced quality of life, loss of independence, and increased mortality [6, 8]. Furthermore, sustaining one vertebral fracture substantially increases the risk of subsequent vertebral and non-vertebral fragility fractures, emphasising their pivotal role in the natural history of osteoporosis [9]. The risk of a subsequent fracture is particularly pronounced during the first one to two years following an initial fragility fracture, a period commonly referred to as “imminent fracture risk”, highlighting the importance of timely secondary fracture prevention [10].

Despite their clinical relevance, vertebral fractures remain substantially underdiagnosed, with many fractures detected only incidentally on imaging. The international IMPACT study demonstrated that approximately one-third of vertebral fractures are missed on routine radiographic assessment, delaying osteoporosis diagnosis and secondary fracture prevention [9]. Failure to recognise vertebral fractures delays the diagnosis of osteoporosis, prevents timely initiation of anti-osteoporotic therapy, and results in missed opportunities for secondary fracture prevention [6, 9].

Although highly effective pharmacological therapies are available, osteoporosis remains markedly undertreated worldwide. Antiresorptive and anabolic agents have consistently been shown to reduce vertebral, non-vertebral, and hip fracture risk in patients with osteoporosis [1]. Recent data from Germany further demonstrate that osteoporosis remains markedly undertreated. In a nationwide analysis, only 30% of individuals with an osteoporotic fracture and/or a confirmed diagnosis of osteoporosis received osteoporosis-specific medication, highlighting the persistent treatment gap in routine clinical care [11]. Nevertheless, the treatment gap has widened over the past decade, with many patients remaining untreated even after sustaining a fragility fracture [2]. Current European guidelines therefore advocate systematic fracture risk assessment, early initiation of evidence-based therapy, and implementation of Fracture Liaison Services to improve secondary fracture prevention [5].

The socioeconomic consequences of osteoporosis are substantial. In Europe, fragility fractures account for tens of billions of euros in annual healthcare expenditure and result in considerable losses in quality-adjusted life years [7]. The economic burden is projected to increase further as populations continue to age, while the persistently large treatment gap is expected to amplify the future demand for healthcare resources [5, 7]. Vertebral fractures contribute considerably to this burden through hospital admissions, advanced imaging, rehabilitation, prolonged disability, and recurrent healthcare utilisation [6].

Despite extensive research on osteoporosis and fragility fractures, contemporary nationwide analyses evaluating long-term hospitalisation trends for vertebral fractures and osteoporosis-related hospitalisations remain limited. Most epidemiological studies have focused on hip fractures, regional cohorts, or relatively short observation periods, whereas comprehensive nationwide analyses spanning more than two decades are scarce. Such data are particularly important in Germany, where demographic ageing is expected to substantially increase the burden of osteoporosis-related hospitalisations and place growing pressure on healthcare systems [5, 7].

Previous nationwide German data have demonstrated increasing inpatient hospitalisation rates for fractures between 2009 and 2019, including increases in thoracic and lumbar vertebral fractures [12]. However, whether these trends persisted beyond the late 2010s and how demographic ageing may affect the future inpatient burden of vertebral fractures remain insufficiently characterised.

Therefore, the present nationwide registry-based study investigated long-term hospitalisation trends for thoracic vertebral fractures (ICD-10-GM S22.0), lumbar vertebral fractures (ICD-10-GM S32.0), and postmenopausal osteoporosis with osteoporotic fracture (ICD-10-GM M80.0; officially classified as “postmenopausal osteoporosis with pathological fracture”) in Germany between 2000 and 2023 using nationwide hospital discharge data from the German Federal Health Monitoring (GBE) database [13]. We evaluated temporal changes in absolute hospitalisation numbers, age-standardised hospitalisation rates, sex-specific trends, in-hospital mortality, and length of hospital stay. Furthermore, we estimated the future inpatient burden through 2050 using official demographic projections [14]. We hypothesised that absolute hospitalisation numbers would increase despite stabilising or declining age-standardised hospitalisation rates and that demographic ageing would lead to a further increase in the future inpatient burden.

## Methods

### Study design and data sources

This nationwide retrospective registry-based study analysed inpatient hospitalisations for thoracic vertebral fractures, lumbar vertebral fractures, and postmenopausal osteoporosis with osteoporotic fracture in Germany between 2000 and 2023. Data were obtained from the German Federal Health Monitoring (Gesundheitsberichterstattung des Bundes, GBE) database, which provides publicly accessible nationwide aggregated hospital discharge statistics compiled by the Federal Statistical Office (Destatis) [13]. The database is based on an annual complete census of inpatient cases in German hospitals, irrespective of health insurance status, with specific exclusions defined by the German Hospital Statistics Ordinance, and includes demographic characteristics, diagnoses coded according to the German Modification of the International Classification of Diseases, 10th Revision (ICD-10-GM), in-hospital mortality, and length of hospital stay. Hospitalisation rates were calculated using annual population estimates provided by the Federal Statistical Office and were age-standardised according to the German standard population of 2022.

### Study population and variables

Hospitalisations were identified using the ICD-10-GM codes for thoracic vertebral fractures (S22.0), lumbar vertebral fractures (S32.0), and postmenopausal osteoporosis with osteoporotic fracture (M80.0) (officially classified as “postmenopausal osteoporosis with pathological fracture”). These diagnoses were selected to capture registry-based hospitalisation trends for thoracic and lumbar vertebral fractures alongside postmenopausal osteoporosis with osteoporotic fracture.

The primary outcome was the annual number of inpatient hospitalisations. Secondary outcomes comprised age-standardised hospitalisation rates, age-specific hospitalisation rates, sex-specific hospitalisation rates, mean length of hospital stay, and in-hospital mortality. Absolute hospitalisation counts were obtained from treatment-location data, whereas age-standardised and age-specific hospitalisation rates were derived from residence-based data provided by the GBE database.

Age-specific hospitalisation rates were evaluated using the age strata available in the GBE database ( < 15, 15–44, 45–64, and ≥65 years) and were reported per 100,000 population within the respective age group. Age-standardised hospitalisation rates were standardised to the German standard population 2022 (“Standardbevölkerung Deutschland 2022”), as provided by the GBE database [13]. Because the GBE database provides aggregated data rather than individual-level patient records, mean or median patient age could not be calculated.

### Demographic projections

Demographic projections of annual hospitalisations through 2050 were performed to estimate the future inpatient burden associated with population ageing. Mean age-specific hospitalisation rates for the age strata <15, 15–44, 45–64, and ≥65 years observed during 2021–2023 were assumed to remain constant and were applied to the corresponding age strata of the official population projections of the German Federal Statistical Office (Destatis). Population estimates were obtained from the main projection scenario (G2L2W2), which represents the official medium population projection for Germany. Projected annual hospitalisations for thoracic vertebral fractures (S22.0) and lumbar vertebral fractures (S32.0) were calculated by multiplying age-specific hospitalisation rates by the corresponding projected population within each age group and summing across all age strata. The projections therefore reflect the expected impact of demographic ageing under the assumption of stable age-specific hospitalisation rates and do not account for potential future changes in disease incidence, diagnostic practices, or treatment strategies. Projected hospitalisations were summarised for 2030, 2040, and 2050.

### Statistical analysis

Descriptive statistics were used to summarise temporal trends in hospitalisation characteristics. Annual hospitalisation counts, age-standardised hospitalisation rates, mean length of hospital stay, and in-hospital mortality were reported as provided by the German Federal Health Monitoring (GBE) database.

Temporal trends in age-standardised hospitalisation rates from 2010 to 2023 were evaluated using joinpoint regression analysis. Joinpoint regression was restricted to this period because continuous annual age-standardised rates were available from 2010 onwards, whereas earlier GBE data were available only for selected years (2000 and 2005). Log-linear segmented regression models were fitted to identify changes in temporal trends. A maximum of two joinpoints was allowed for each model, with stepwise model selection used to determine the number of joinpoints. Annual percent change (APC) with corresponding 95% confidence intervals (CIs) was calculated for each linear segment, and average annual percent change (AAPC) was estimated to summarise the overall trend across the study period. Statistical significance was defined as a two-sided *p* value < 0.05.

All statistical analyses were performed using R version 4.6.1 (R Foundation for Statistical Computing, Vienna, Austria). Temporal trend analyses were conducted using the joinpointR package (version 1.0.0) [15].

## Results

### Overview of nationwide hospitalisation trends

**Fig. 1:**
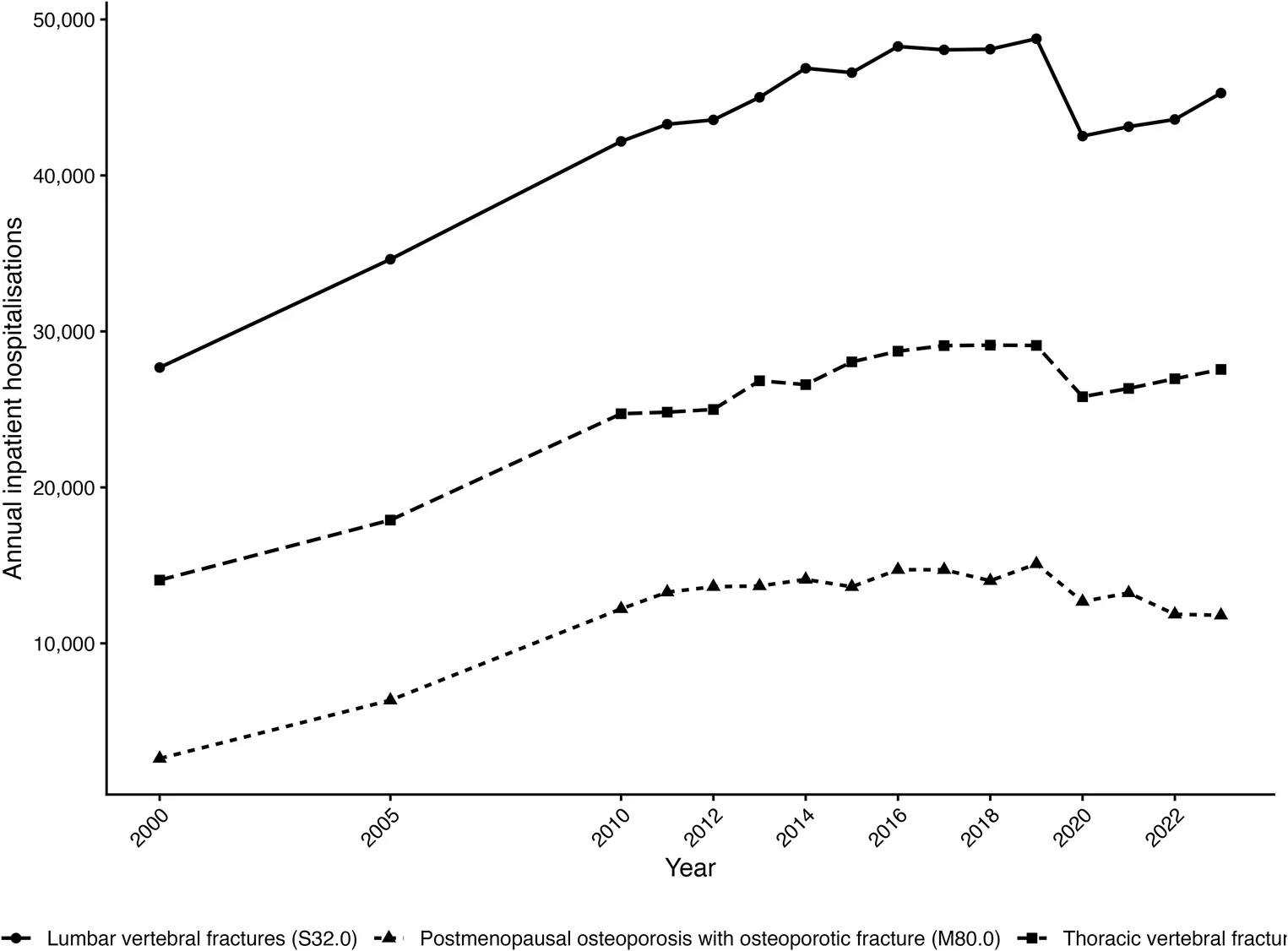
Nationwide trends in annual inpatient hospitalisations of vertebral fractures and postmenopausal osteoporosis with osteoporotic fracture in Germany. Absolute numbers of hospitalisations for thoracic vertebral fractures (ICD-10-GM S22.0), lumbar vertebral fractures (ICD-10-GM S32.0), and postmenopausal osteoporosis with osteoporotic fracture (ICD-10-GM M80.0) are shown for the available observation years between 2000 and 2023. Data were obtained from the German Federal Health Monitoring System. The marked decrease in 2020 was observed across all three diagnostic groups, followed by partial recovery through 2023. ICD-10-GM, International Classification of Diseases, 10th Revision, German Modification

Between 2000 and 2023, annual inpatient hospitalisations increased substantially for all investigated diagnoses in Germany (Fig. 1). Hospitalisations for thoracic vertebral fractures (S22.0) increased from 14,057 cases in 2000 to 27,563 cases in 2023, while lumbar vertebral fractures (S32.0) increased from 27,683 to 45,280 cases. The largest relative increase was observed for postmenopausal osteoporosis with osteoporotic fracture (M80.0), with hospitalisations increasing from 2,617 to 11,800 cases over the study period.

All three diagnoses reached their highest hospitalisation numbers during the late 2010s. Thoracic vertebral fractures peaked in 2018 (29,119 cases), whereas lumbar vertebral fractures and postmenopausal osteoporosis with osteoporotic fracture reached their highest numbers in 2019 (48,763 and 15,095 cases, respectively). A pronounced decline in hospitalisations was observed across all diagnoses in 2020, followed by partial recovery through 2023. However, hospitalisation numbers remained below their respective pre-pandemic peak levels.

### Hospitalisation characteristics and in-hospital outcomes

Women accounted for the majority of hospitalisations throughout the study period (Table 1). In 2023, women represented 62.2% of thoracic vertebral fracture admissions, 60.3% of lumbar vertebral fracture admissions, and 82.0% of admissions for postmenopausal osteoporosis with osteoporotic fracture.

**Table 1:** Characteristics of hospitalisations for thoracic vertebral fractures (ICD-10-GM S22.0), lumbar vertebral fractures (ICD-10-GM S32.0), and postmenopausal osteoporosis with osteoporotic fracture (ICD-10-GM M80.0) in Germany

Characteristic	Thoracic vertebral fractures (S22.0)	Lumbar vertebral fractures (S32.0)	Postmenopausal osteoporosis with osteoporotic fracture (M80.0)
Hospitalisations, 2000	14,057	27,683	2,617
Hospitalisations, 2010	24,721	42,181	12,212
Hospitalisations, 2019	29,100	48,763	15,095
Hospitalisations, 2023	27,563	45,280	11,800
Relative change, 2000–2023 (%)	+96.1	+63.6	+350.9
Female share of hospitalisations, 2023 (%)	62.2	60.3	82.0
Mean length of stay, 2000 (days)	15.4	16.1	16.5
Mean length of stay, 2023 (days)	10.4	10.4	11.7
In-hospital mortality, 2000 (%)	3.4	2.6	0.6
In-hospital mortality, 2023 (%)	4.9	4.9	1.3

Data are presented for selected observation years to illustrate long-term changes in annual hospitalisation numbers, sex distribution, mean length of hospital stay (LOS), and in-hospital mortality. Relative changes refer to the period from 2000 to 2023. Data were obtained from the German Federal Health Monitoring System (GBE)

Age-specific hospitalisation rates differed substantially across age groups (Supplementary Table S3). Among individuals aged ≥65 years, hospitalisation rates for thoracic vertebral fractures increased from 48 per 100,000 in 2000 to 107 per 100,000 in 2023, while rates for lumbar vertebral fractures increased from 111 to 183 per 100,000. In contrast, hospitalisation rates in younger age groups remained substantially lower and were generally stable or declined over the observation period. For postmenopausal osteoporosis with osteoporotic fracture, the hospitalisation rate among individuals aged ≥65 years increased from 17 per 100,000 in 2000 to 75 per 100,000 in 2015 and subsequently declined to 60 per 100,000 in 2023 (Supplementary Table S3).

Mean length of hospital stay declined continuously across all investigated diagnoses. Between 2000 and 2023, the average hospital stay decreased from 15.4 to 10.4 days for thoracic vertebral fractures, from 16.1 to 10.4 days for lumbar vertebral fractures, and from 16.5 to 11.7 days for postmenopausal osteoporosis with osteoporotic fracture.

In 2023, mortality rates were 4.9% for thoracic vertebral fractures, 4.9% for lumbar vertebral fractures, and 1.3% for postmenopausal osteoporosis with osteoporotic fracture.

### Age-standardised and sex-specific hospitalisation rates

**Fig. 2:**
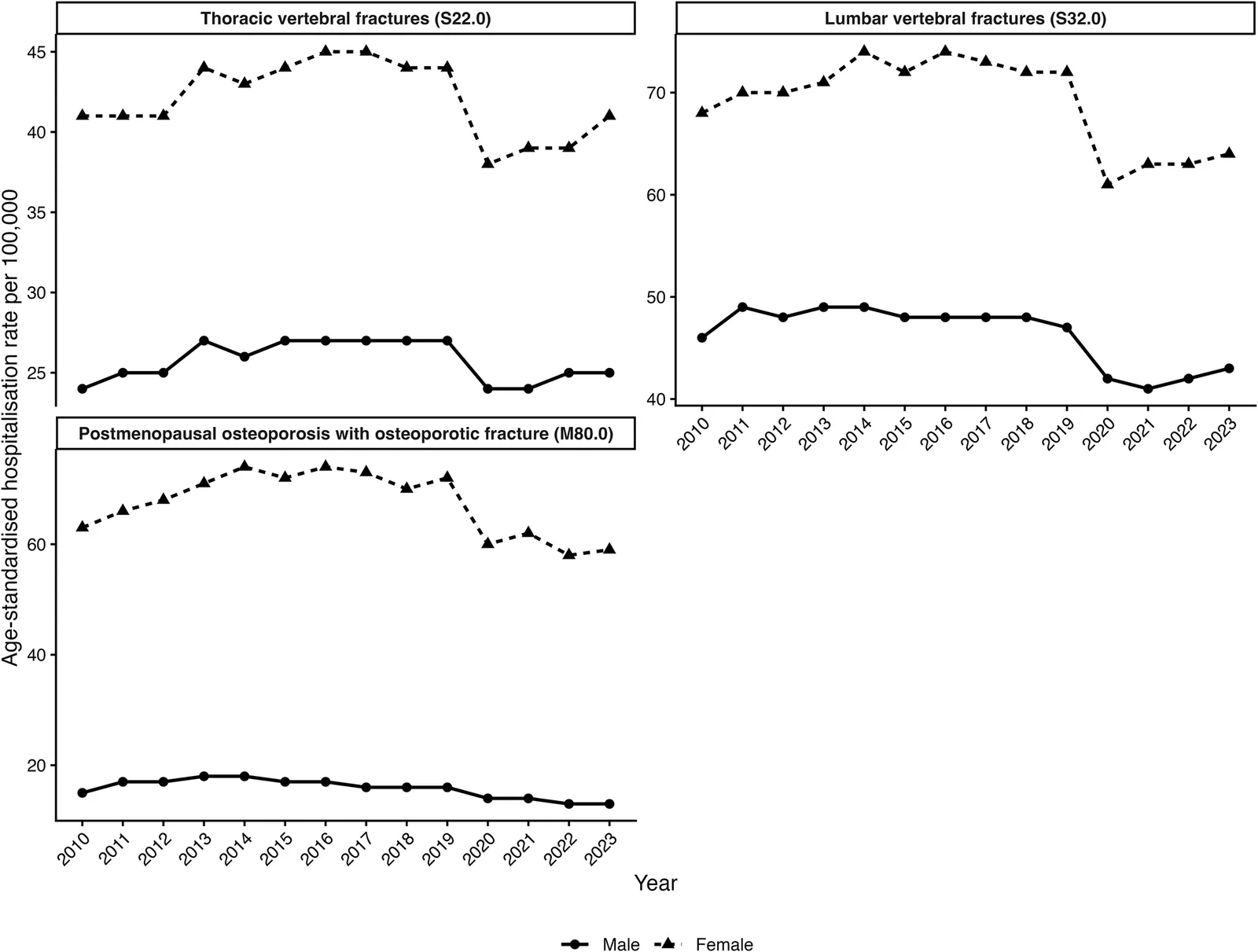
Age-standardised hospitalisation rates for thoracic vertebral fractures (ICD-10-GM S22.0), lumbar vertebral fractures (ICD-10-GM S32.0), and postmenopausal osteoporosis with osteoporotic fracture (ICD-10-GM M80.0) in Germany between 2010 and 2023. Hospitalisation rates are presented per 100,000 population according to the German standard population 2022 and are shown separately for men and women. All three conditions demonstrated increasing hospitalisation rates until the mid-to-late 2010s followed by a subsequent decline, while absolute hospitalisation numbers remained substantially higher than at the beginning of the observation period

Age-standardised hospitalisation rates demonstrated a temporal pattern distinct from the corresponding absolute hospitalisation numbers (Fig. 2). Whereas absolute hospitalisations increased substantially over the study period, age-standardised rates increased until the mid-to-late 2010s before declining across all three diagnoses.

For thoracic vertebral fractures, age-standardised hospitalisation rates increased from 33 per 100,000 population in 2010 to 37 per 100,000 in 2016–2017 before declining to 33 per 100,000 in 2023. Lumbar vertebral fractures showed a similar pattern, increasing from 58 to 62 per 100,000 population before decreasing to 54 per 100,000 in 2023. Postmenopausal osteoporosis with osteoporotic fracture demonstrated the greatest temporal variation, increasing from 63 to 74 per 100,000 women before declining to 59 per 100,000 in 2023.

Sex-specific analyses consistently demonstrated higher hospitalisation rates among women than men for all investigated diagnoses. This difference was particularly pronounced for postmenopausal osteoporosis with osteoporotic fracture, whereas sex differences were less marked for thoracic and lumbar vertebral fractures.

### Joinpoint trend analysis

**Fig. 3:**
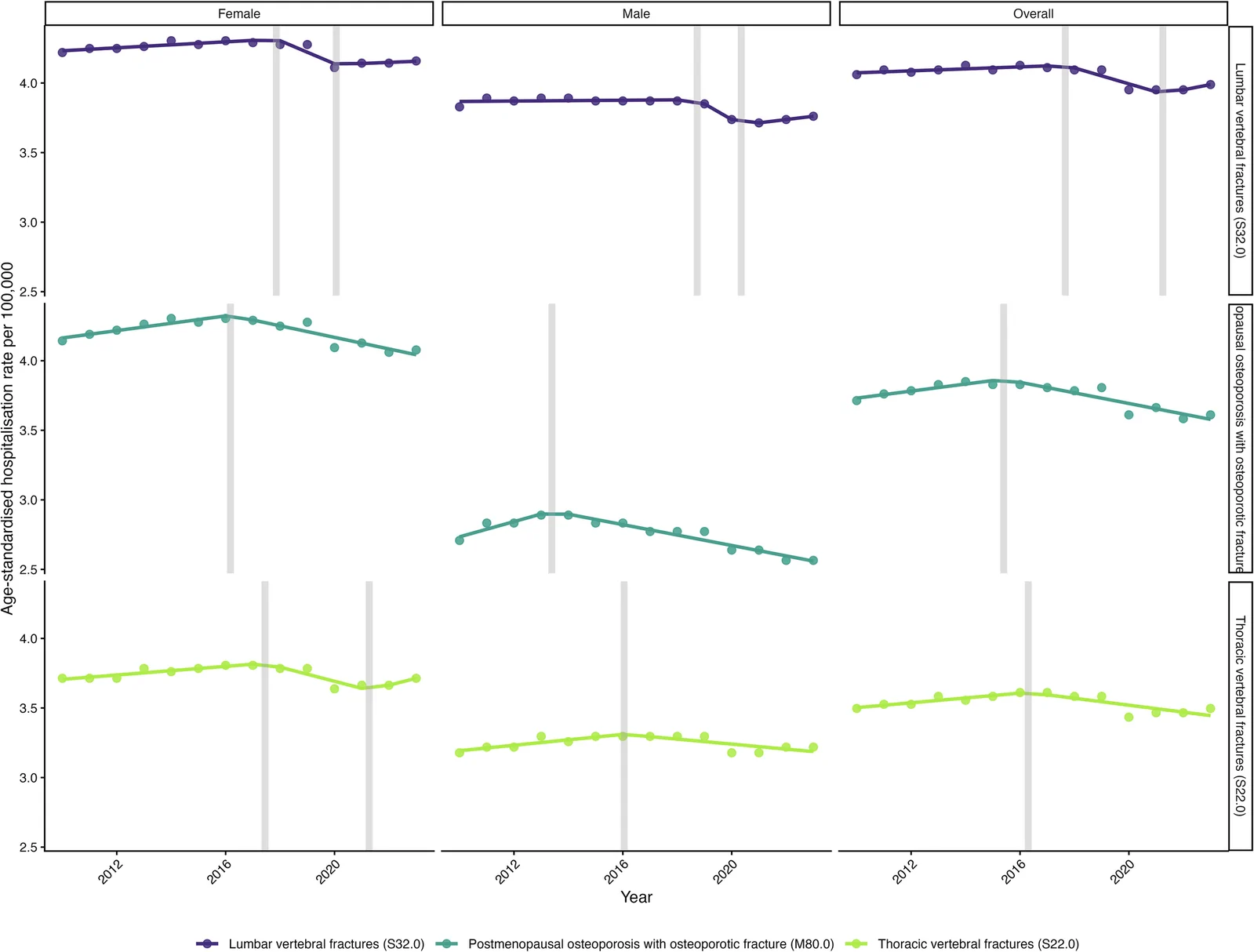
Joinpoint regression analysis of age-standardised hospitalisation rates for thoracic vertebral fractures (ICD-10-GM S22.0), lumbar vertebral fractures (ICD-10-GM S32.0), and postmenopausal osteoporosis with osteoporotic fracture (ICD-10-GM M80.0) in Germany between 2010 and 2023. Solid lines represent the fitted joinpoint regression models, while circles indicate the observed age-standardised hospitalisation rates. Joinpoints indicate statistically significant changes in temporal trends. Age-standardisation was performed using the German standard population 2022

Joinpoint regression analysis confirmed significant changes in temporal hospitalisation trends for all investigated diagnoses (Fig. 3, Table 2).

**Table 2:** Joinpoint regression analysis of age-standardised hospitalisation rates for thoracic vertebral fractures (S22.0), lumbar vertebral fractures (S32.0), and postmenopausal osteoporosis with osteoporotic fracture (M80.0) in Germany between 2010 and 2023

Diagnosis	Time interval	APC (%)	95% CI	*P* value
**S22.0**	2010–2016	+1.75	0.15 to 3.38	0.035
	2016–2023	−2.46	−3.99 to −0.90	0.006
	**AAPC**	**−0.44**	−0.99 to 0.11	0.10
**S32.0**	2010–2018	+0.72	−0.23 to 1.68	0.12
	2018–2021	−5.56	−8.12 to −2.93	0.001
	2021–2023	+3.85	−4.80 to 13.28	0.35
	**AAPC**	**−0.65**	−1.13 to −0.17	0.01
**M80.0**	2010–2015	+2.57	0.33 to 4.85	0.03
	2015–2023	−3.73	−5.10 to −2.36	<0.001
	**AAPC**	**−1.17**	−1.78 to −0.57	<0.001

Annual percent change (APC), average annual percent change (AAPC), 95% confidence intervals (CI), and corresponding *p*-values are presented for the overall population. Sex-specific analyses are provided in Supplementary Table S1

For thoracic vertebral fractures, age-standardised hospitalisation rates increased significantly between 2010 and 2016 (annual percent change [APC] +1.75%, 95% confidence interval [CI] 0.15 to 3.38; *p* = 0.035), followed by a significant decline between 2016 and 2023 (APC − 2.46%, 95% CI − 3.99 to −0.90; *p* = 0.006). Overall, no significant long-term change was observed (average annual percent change [AAPC] −0.44%, 95% CI − 0.99 to 0.11; *p* = 0.10).

Lumbar vertebral fractures remained stable until 2018, followed by a significant decline between 2018 and 2021 (APC − 5.56%, 95% CI − 8.12 to −2.93; *p* = 0.001). Although hospitalisation rates increased slightly thereafter, this change did not reach statistical significance (APC + 3.85%, 95% CI − 4.80 to 13.28; *p* = 0.35). Overall, lumbar vertebral fractures demonstrated a significant long-term reduction in hospitalisation rates (AAPC − 0.65%, 95% CI − 1.13 to −0.17; *p* = 0.01).

Postmenopausal osteoporosis with osteoporotic fracture exhibited the most pronounced temporal changes. Hospitalisation rates increased significantly between 2010 and 2015 (APC + 2.57%, 95% CI 0.33 to 4.85; *p* = 0.03), followed by a continuous decline between 2015 and 2023 (APC − 3.73%, 95% CI − 5.10 to −2.36; *p* < 0.001). Consequently, this diagnosis demonstrated the largest overall reduction in age-standardised hospitalisation rates (AAPC − 1.17%, 95% CI − 1.78 to −0.57; *p* < 0.001).

Sex-specific joinpoint analyses demonstrated similar temporal patterns in men and women, although hospitalisation rates remained consistently higher among women across all investigated diagnoses (Supplementary Table S1).

### Demographic projections through 2050

**Fig. 4:**
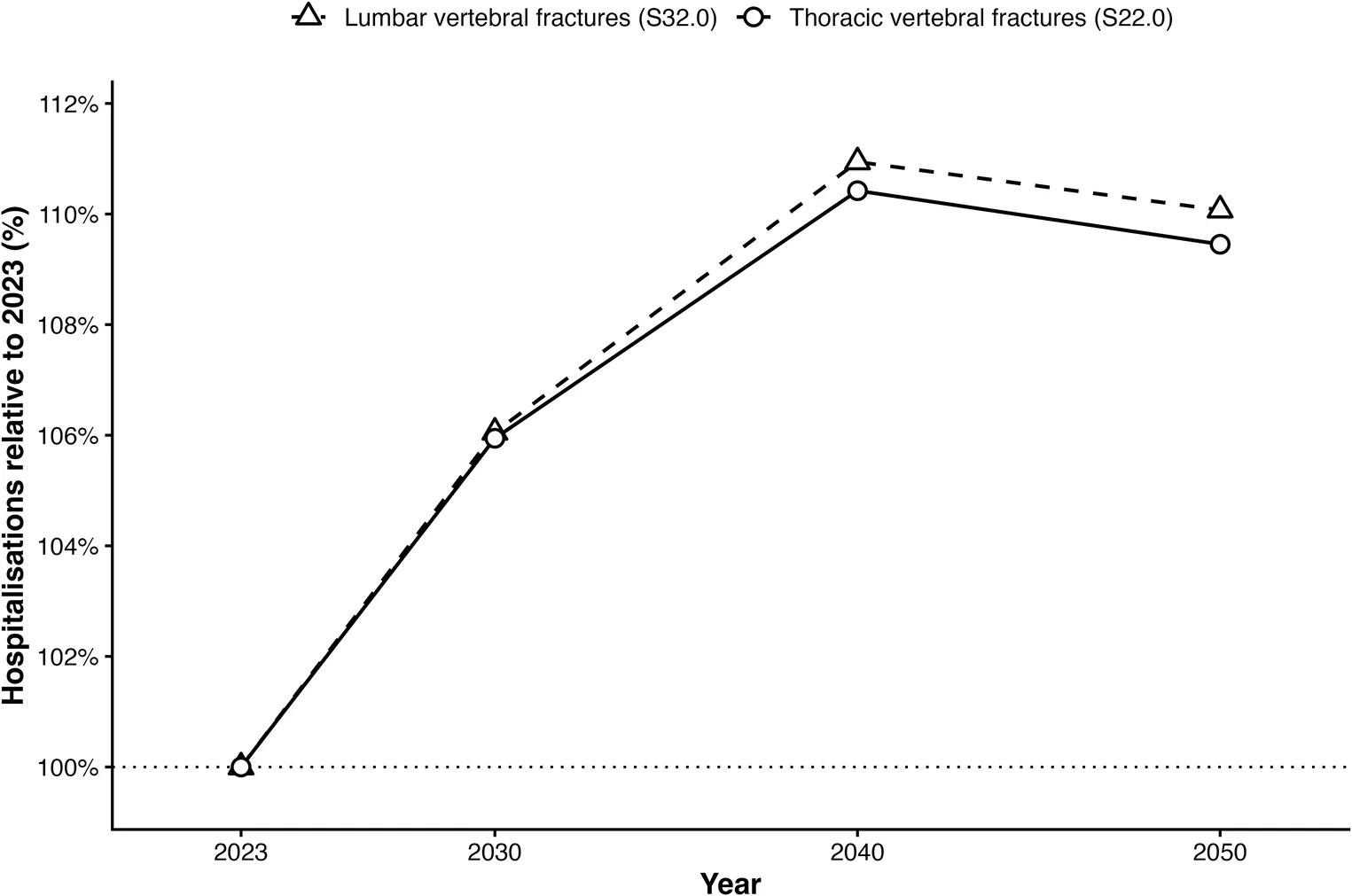
Demographic projections of annual hospitalisations for thoracic and lumbar vertebral fractures in Germany through 2050. Projected annual hospitalisations for thoracic vertebral fractures (ICD-10-GM S22.0) and lumbar vertebral fractures (ICD-10-GM S32.0) were estimated by applying the mean age-specific hospitalisation rates observed during 2021–2023 to the official population projections of the German Federal Statistical Office (Destatis; main scenario G2L2W2), assuming constant age-specific hospitalisation rates over time

Assuming constant age-specific hospitalisation rates observed during 2021–2023, the demographic projection indicated an increase in the future inpatient burden of thoracic and lumbar vertebral fractures in Germany through 2050 (Fig. 4). Annual hospitalisations for thoracic vertebral fractures are projected to increase from 27,563 cases in 2023 to approximately 30,200 cases in 2050, corresponding to an increase of 9.5%. Similarly, hospitalisations for lumbar vertebral fractures are projected to rise from 45,280 to ~49,800 cases ( + 10.1%).

These projections indicate that, despite stabilising or declining age-standardised hospitalisation rates observed during the most recent decade, the absolute number of hospitalisations may remain high and increase further under the assumed demographic scenario.

## Discussion

### Principal findings

In this nationwide registry-based study spanning 24 years, we observed marked increases in absolute hospitalisations for thoracic vertebral fractures, lumbar vertebral fractures, and postmenopausal osteoporosis with osteoporotic fracture in Germany. In contrast, age-standardised hospitalisation rates increased until the mid-to-late 2010s before stabilising or declining, with significant long-term reductions observed for lumbar vertebral fractures and postmenopausal osteoporosis with osteoporotic fracture. Mean length of hospital stay decreased continuously, whereas in-hospital mortality did not show a corresponding decline. Importantly, despite recent stabilisation or declines in age-standardised hospitalisation rates, demographic modelling indicated an approximately 10% increase in annual inpatient hospitalisations for thoracic and lumbar vertebral fractures by 2050 under the assumed demographic scenario.

Taken together, these findings demonstrate that recent declines in age-standardised hospitalisation rates are unlikely to fully offset the growing healthcare burden associated with demographic ageing, as the growing older population is expected to remain a major driver of inpatient care over the coming decades. This interpretation is further supported by the age-specific analysis, which demonstrated that hospitalisation rates were substantially higher among individuals aged ≥65 years than in younger age groups, particularly for thoracic and lumbar vertebral fractures (Supplementary Table S3). However, because S22.0 and S32.0 are not specific to osteoporotic fractures, these age-specific patterns should be interpreted as reflecting the age distribution of vertebral fracture hospitalisations rather than osteoporosis-specific fracture incidence.

Our findings extend previous nationwide German data reported by Rupp et al., who observed increases in inpatient thoracic and lumbar spine fracture rates of 32% and 21%, respectively, between 2009 and 2019 [12]. A subsequent nationwide analysis by Lang et al. similarly reported a 45.6% increase in documented hospitalised vertebral fractures overall between 2009 and 2019 [16]. By extending the observation period through 2023, the present study demonstrates that these earlier increases did not continue uniformly: age-standardised hospitalisation rates subsequently stabilised or declined, with joinpoint analyses identifying trend reversals in 2016 for thoracic and 2018 for lumbar vertebral fractures. In addition, our analysis incorporates postmenopausal osteoporosis with osteoporotic fracture, long-term trends in inpatient outcomes, and demographic projections of vertebral fracture hospitalisations through 2050.

Importantly, the observed trends represent changes in inpatient hospitalisations rather than changes in the overall incidence of vertebral fractures. A substantial proportion of vertebral fractures remain clinically silent or are managed without hospital admission and are therefore not captured by the present data. Moreover, the stabilisation or decline in hospitalisation rates after the identified joinpoints may reflect several factors, including changes in diagnostic and treatment pathways, the use and setting of vertebral augmentation procedures such as vertebroplasty or kyphoplasty, outpatient management, hospital admission thresholds, or osteoporosis treatment. Accordingly, the observed trend reversals should not be interpreted as direct evidence of a corresponding decline in the underlying incidence of vertebral fractures.

These results are consistent with previous European and global studies showing that demographic ageing has become the principal driver of the increasing fracture burden despite stabilising or declining age-standardised rates. They also align with previous projections from Germany. Using a demographic simulation model, Bleibler et al. projected a marked increase in osteoporosis-attributable fractures and associated healthcare costs through 2050, largely driven by demographic ageing rather than changes in fracture risk [17]. While that study estimated the future burden using simulation modelling, our nationwide analysis provides empirical hospitalisation data over a 24-year period and shows that the observed inpatient trends are consistent with the demographic patterns anticipated by these projections. Furthermore, our demographic projections indicate that this increasing inpatient burden is likely to continue despite recent stabilisation or declines in age-standardised hospitalisation rates.

These findings are further supported by European and national data. An EU-wide assessment estimated 3.5 million incident fragility fractures and healthcare costs of €37 billion in 2010 and projected a substantial future increase driven primarily by demographic change [5]. Similarly, a nationwide German claims-based analysis estimated that approximately 3.6 million individuals had a documented diagnosis of osteoporosis in 2016 and concluded that demographic ageing is likely to substantially increase the number of patients with osteoporosis and osteoporotic fractures over the coming decades [18]. Likewise, the recent German BEST-2 study estimated that 5.87 million individuals aged ≥50 years were living with osteoporosis in 2018 and projected a continued increase in disease burden as the population ages [11]. At the global level, age-standardised incidence, prevalence, and disability rates of vertebral fractures have declined over recent decades, whereas absolute case numbers have continued to rise owing to population ageing [19]. Our nationwide analysis extends these observations by demonstrating the same divergence between absolute and age-standardised hospitalisation burden in Germany over a 24-year period while additionally providing projections of future inpatient demand through 2050.

### Temporal changes and the decline after the late 2010s

The decline in age-standardised hospitalisation rates after 2015–2018 may reflect several overlapping developments. Improvements in osteoporosis awareness, fracture-risk assessment, pharmacological prevention, fall prevention, and coordinated secondary prevention may have contributed to lower hospitalisation risk. Effective antiresorptive and anabolic therapies reduce vertebral fracture risk substantially in appropriately selected high-risk patients, and European guidance recommends systematic fracture-risk assessment and treatment after fragility fractures [20]. Nevertheless, the aggregated nature of the GBE data does not permit direct assessment of treatment exposure, adherence, outpatient management, or causal attribution. The observed decline should therefore not be interpreted as direct evidence of improved osteoporosis treatment.

Moreover, persistent undertreatment remains a major concern. Recent German data from the BEST-2 study estimated that ~5.87 million individuals aged 50 years or older were affected by osteoporosis in 2018, while only 30% of those with an osteoporotic fracture and/or a confirmed osteoporosis diagnosis received osteoporosis-specific medication. The same study found that osteoporosis was diagnosed in only 39% of individuals who had sustained an osteoporotic fracture, underscoring persistent deficiencies in both recognition and treatment [11]. These findings are consistent with broader European evidence demonstrating a substantial post-fracture treatment gap despite the availability of effective therapies and established clinical guidance [2, 7]. Therefore, declining age-standardised hospitalisation rates should not be interpreted as evidence that osteoporosis care is adequate. They may also reflect changes in admission thresholds, coding practices, outpatient treatment pathways, or the management of less severe fractures.

The pronounced reduction in 2020 across all diagnostic groups is likely related to the COVID-19 pandemic. During the pandemic, reductions in hospital admissions, diagnostic procedures, osteoporosis screening, and healthcare-seeking were reported internationally. A recent Lancet review reported that DXA utilisation declined by more than 50% globally during the COVID-19 pandemic [21]. Because the present data do not distinguish reduced fracture occurrence from reduced presentation or altered admission practices, the 2020 decline should be interpreted primarily as a healthcare-utilisation disruption rather than a sudden epidemiological improvement.

### Sex-specific patterns

Women consistently demonstrated higher hospitalisation rates than men, particularly for postmenopausal osteoporosis with osteoporotic fracture. This finding is biologically and epidemiologically plausible because accelerated bone loss after menopause substantially increases skeletal fragility in older women. Global analyses show that although vertebral fracture rates may be higher in men at younger ages, women exceed men in prevalence and disability burden at older ages, when postmenopausal osteoporosis becomes dominant [19].

The sex difference was less pronounced for S22.0 and S32.0 than for M80.0. This likely reflects the broader aetiological spectrum captured by traumatic vertebral fracture codes, which may include fractures related to high-energy trauma, falls, malignancy, and other non-osteoporotic mechanisms. In contrast, M80.0 explicitly identifies postmenopausal osteoporosis with osteoporotic fracture and is therefore expected to be strongly female-predominant.

### Clinical burden, underdiagnosis, and secondary prevention

The persistent absolute burden observed in this study is clinically relevant because vertebral fractures are associated with pain, disability, deformity, reduced quality of life, subsequent fractures, and mortality. The QUALEFFO validation study demonstrated substantial impairment in pain, physical functioning, and social functioning among patients with vertebral fractures [8]. Beyond their clinical consequences, vertebral fractures also impose a considerable economic burden. Previous modelling from Germany demonstrated that vertebral fractures represent the second largest contributor to osteoporosis-related healthcare costs after hip fractures, with hospital care and long-term care accounting for the largest proportion of lifetime fracture-related costs [4]. Vertebral fractures are also frequently clinically silent or overlooked. In the multinational IMPACT study, comparison of local and central radiographic readings yielded a false-negative rate of 34%, and approximately three-quarters of vertebral fractures failed to come to immediate clinical attention [9]. This underrecognition is clinically important because a prevalent vertebral fracture markedly increases the risk of subsequent vertebral and other fragility fractures.

The present hospitalisation data therefore likely represent only the more clinically severe end of the vertebral fracture spectrum. They do not capture asymptomatic fractures, fractures identified incidentally, or cases managed exclusively in outpatient care. Consequently, a decline in inpatient rates does not necessarily imply a comparable decline in the underlying population prevalence of vertebral fractures. Accordingly, the true population burden of osteoporotic vertebral fractures is likely to be substantially greater than reflected by inpatient hospitalisation data alone.

The findings support stronger secondary fracture prevention. Fracture Liaison Services offer a structured approach to identifying patients with fragility fractures, assessing fracture risk, and initiating appropriate treatment. Previous evaluations have shown that such services improve treatment uptake and can reduce recurrent fractures while remaining cost-effective [5, 21]. Given the large number of hospitalisations identified in this study, inpatient treatment episodes represent important opportunities for osteoporosis assessment, vertebral fracture recognition, and coordinated post-discharge care.

### Length of stay and in-hospital mortality

Mean length of stay declined substantially for all diagnoses over the study period. This trend may reflect more efficient diagnostic pathways, earlier mobilisation, advances in pain management and fracture care, pressure to reduce inpatient duration, and a broader shift from prolonged hospitalisation toward outpatient or rehabilitation-based management. However, because the GBE database does not provide treatment-level information, the contribution of specific clinical or organisational changes cannot be quantified.

In-hospital mortality did not show a corresponding decline despite shorter hospital stays and increasing absolute case numbers. This may suggest that reductions in length of stay were not accompanied by an obvious deterioration in short-term inpatient survival. Nevertheless, in-hospital mortality is an incomplete outcome because it excludes deaths after discharge and is sensitive to discharge practices, transfer to rehabilitation or long-term care, and changes in admission thresholds. The mortality values should therefore not be interpreted as comprehensive fracture-associated mortality.

### Demographic projections through 2050

The demographic projection extends the historical analysis by estimating future inpatient hospitalisations under the assumption of stable age-specific hospitalisation rates and the official German demographic scenario. Assuming that mean age-specific hospitalisation rates from 2021–2023 remain unchanged, annual hospitalisations were projected to increase by ~9.5% for thoracic vertebral fractures and 10.1% for lumbar vertebral fractures by 2050. These findings are compatible with European burden models demonstrating that demographic change can increase absolute fracture numbers even when standardised rates stabilise or decline [5]. They also complement recent global projections that anticipate continued changes in vertebral fracture burden through 2050, although the global study used a different ARIMA-based methodology and projected declining age-standardised rates [19].

The projections should be interpreted as scenario-based estimates rather than forecasts of future disease incidence. They isolate the effect of the official medium population projection of the German Federal Statistical Office (Destatis; G2L2W2) under the assumption of unchanged age-specific hospitalisation rates [14]. They do not account for future changes in osteoporosis prevalence, pharmacological treatment, screening, fall prevention, diagnostic coding, admission thresholds, outpatient substitution, or healthcare capacity. Their value lies in illustrating how projected demographic change may influence the absolute inpatient burden under the assumption of stable age-specific hospitalisation rates, despite recent declines in standardised rates.

### Strengths and limitations

The principal strengths of this study include its nationwide coverage, 24-year observation period, use of age-standardised and sex-specific hospitalisation rates, and application of joinpoint regression to identify changes in temporal trends. Furthermore, combining historical nationwide data with demographic projections based on the official German population scenario (G2L2W2) provides a clinically and health-policy-relevant assessment of both past trends and the future inpatient burden of vertebral fractures.

Several limitations should be acknowledged. First, the study was based on aggregated administrative data, precluding patient-level adjustment for comorbidities, fracture severity, bone mineral density, trauma mechanism, treatment, recurrence, or medication exposure. Second, ICD-10-GM coding may be affected by diagnostic inaccuracies and temporal changes in diagnostic awareness, imaging utilisation, documentation, coding practices, reimbursement, and case ascertainment over the 24-year observation period. Consequently, part of the observed temporal variation in hospitalisation rates may reflect changes in diagnostic and coding practices rather than changes in the underlying fracture burden alone. Moreover, S22.0 and S32.0 are not specific to osteoporotic fractures and may also include traumatic or pathological fractures of other aetiologies. Third, hospitalisations rather than individual patients were analysed, and repeated admissions could not be identified. Fourth, the database captures inpatient care only and therefore underestimates the total burden of vertebral fractures, particularly because many vertebral fractures remain clinically silent or are managed in the outpatient setting. Fifth, the demographic projections assume stable age-specific hospitalisation rates and are based on a single official population scenario; therefore, they should be interpreted as projections rather than precise forecasts. Finally, the observational design does not permit causal inference regarding the factors underlying the observed temporal trends.

## Conclusions

This nationwide analysis demonstrates that the absolute inpatient burden of vertebral fractures and postmenopausal osteoporosis with osteoporotic fracture increased substantially in Germany between 2000 and 2023, while age-standardised hospitalisation rates declined after the mid-to-late 2010s. The divergence between absolute and age-standardised trends highlights the potential importance of demographic ageing for future inpatient healthcare utilisation. Despite recent rate reductions, demographic projections indicate that inpatient hospitalisations for thoracic and lumbar vertebral fractures may increase further by 2050. These findings underscore the need for timely osteoporosis diagnosis, systematic secondary fracture prevention, wider implementation of Fracture Liaison Services, and proactive healthcare planning to address the future inpatient burden associated with vertebral fractures in an ageing population.

## Supplementary information

Table S1

Table S2

Table S3

## Data Availability

The data used in this study are publicly available aggregated administrative data from the German Federal Health Monitoring System (GBE-Bund) [13]. No individual-level patient data were used.
