# Supplementary material for: Nationwide trends and demographic projections of hospitalisations for vertebral fractures and postmenopausal osteoporosis in Germany: a 24-year registry-based study
Source: Arch Osteoporos. 2026 Oct 30;21(1):154. doi: 10.1007/s11657-026-01796-5 (PMC13722723; doi:10.1007/s11657-026-01796-5)
Supplement: Supplementary file 1 [file 11657_2026_1796_MOESM1_ESM.docx]

**Nationwide Trends and Demographic Projections of Hospitalisations for Vertebral Fractures and Postmenopausal Osteoporosis in Germany: A 24-Year Registry-Based Study**

Houmam Anees^1,2^*****, Khaled Qadourah^1^, Goran Georgievski^1^, Christian Heiss^1,2,3^ and Thaqif El Khassawna^2,4^

^1^Department of Trauma, Hand and Reconstructive Surgery, Faculty of Medicine, Justus-Liebig-University of Giessen, 35392 Giessen, Germany

^2^Experimental Trauma Surgery, Faculty of Medicine, Justus-Liebig-University of Giessen, 35392 Giessen, Germany

^3^Biruni University, Istanbul, Türkiye

^4^School of Pharmacy, The University of Jordan, Amman 11942, Jordan

***Corresponding Author:** [Houmam.Anees@chiru.med.uni-giessen.de](mailto:Houmam.Anees@chiru.med.uni-giessen.de) (H. Anees).

| **Diagnosis** | **Sex** | **Joinpoints** | **Time interval** | **APC (%)** | **95% CI** | **AAPC (%)** |
| --- | --- | --- | --- | --- | --- | --- |
| **Thoracic vertebral fractures (S22.0)** | Male | 1 | 2010–2016 | +1.97 | 0.51 to 3.45 | −0.05 |
|  |  |  | 2016–2023 | −1.78 | −3.18 to −0.35 |  |
|  | Female | 2 | 2010–2017 | +1.57 | 0.50 to 2.65 | +0.06 |
|  |  |  | 2017–2021 | −4.96 | −7.83 to −1.99 |  |
|  |  |  | 2021–2023 | +5.13 | −4.61 to 15.86 |  |
| **Lumbar vertebral fractures (S32.0)** | Male | 2 | 2010–2019 | +0.15 | −0.42 to 0.72 | **−0.82*** |
|  |  |  | 2019–2020 | −10.64 | −16.03 to −4.90 |  |
|  |  |  | 2020–2023 | +2.41 | −0.73 to 5.65 |  |
|  | Female | 2 | 2010–2018 | +1.08 | 0.09 to 2.08 | **−0.58*** |
|  |  |  | 2018–2020 | −7.96 | −12.01 to −3.71 |  |
|  |  |  | 2020–2023 | +0.79 | −3.65 to 5.43 |  |
| **Postmenopausal osteoporosis with osteoporotic fracture (M80.0)** | Male | 1 | 2010–2013 | +5.62 | 2.25 to 9.11 | **−1.34*** |
|  |  |  | 2013–2023 | −3.68 | −4.44 to −2.90 |  |
|  | Female | 1 | 2010–2016 | +2.68 | 1.05 to 4.34 | **−0.93*** |
|  |  |  | 2016–2023 | −4.07 | −5.60 to −2.53 |  |

** Statistically significant average annual percent change (p < 0.05).*

***Supplementary Table S1.*** *Sex-specific joinpoint regression analysis of age-standardised hospitalisation rates for thoracic vertebral fractures (ICD-10-GM S22.0), lumbar vertebral fractures (ICD-10-GM S32.0), and postmenopausal osteoporosis with osteoporotic fracture (ICD-10-GM M80.0) in Germany between 2010 and 2023. The table presents the number of identified joinpoints, the corresponding time intervals, annual percent change (APC), 95% confidence intervals (CI), and average annual percent change (AAPC) separately for males and females. Positive APC values indicate increasing hospitalisation rates, whereas negative values indicate declining hospitalisation rates.*

***Abbreviations: APC, annual percent change; AAPC, average annual percent change; CI, confidence interval.***
