# Supplementary material for: Nationwide trends and demographic projections of hospitalisations for vertebral fractures and postmenopausal osteoporosis in Germany: a 24-year registry-based study
Source: Arch Osteoporos. 2026 Oct 30;21(1):154. doi: 10.1007/s11657-026-01796-5 (PMC13722723; doi:10.1007/s11657-026-01796-5)
Supplement: Supplementary file 2 [file 11657_2026_1796_MOESM2_ESM.docx]

**Nationwide Trends and Demographic Projections of Hospitalisations for Vertebral Fractures and Postmenopausal Osteoporosis in Germany: A 24-Year Registry-Based Study**

Houmam Anees^1,2^*****, Khaled Qadourah^1^, Goran Georgievski^1^, Christian Heiss^1,2,3^ and Thaqif El Khassawna^2,4^

^1^Department of Trauma, Hand and Reconstructive Surgery, Faculty of Medicine, Justus-Liebig-University of Giessen, 35392 Giessen, Germany

^2^Experimental Trauma Surgery, Faculty of Medicine, Justus-Liebig-University of Giessen, 35392 Giessen, Germany

^3^Biruni University, Istanbul, Türkiye

^4^School of Pharmacy, The University of Jordan, Amman 11942, Jordan

***Corresponding Author:** [Houmam.Anees@chiru.med.uni-giessen.de](mailto:Houmam.Anees@chiru.med.uni-giessen.de) (H. Anees).

| **Year** | **Thoracic vertebral fractures (S22.0)** | **Lumbar vertebral fractures (S32.0)** | **Postmenopausal osteoporosis with osteoporotic fracture (M80.0)** |
| --- | --- | --- | --- |
| 2010 | 33 | 58 | 63 |
| 2011 | 34 | 60 | 66 |
| 2012 | 34 | 59 | 68 |
| 2013 | 36 | 60 | 71 |
| 2014 | 35 | 62 | 74 |
| 2015 | 36 | 60 | 72 |
| 2016 | 37 | 62 | 74 |
| 2017 | 37 | 61 | 73 |
| 2018 | 36 | 60 | 72 |
| 2019 | 36 | 60 | 72 |
| 2020 | 31 | 52 | 60 |
| 2021 | 32 | 52 | 62 |
| 2022 | 32 | 52 | 58 |
| 2023 | 33 | 54 | 59 |

***Supplementary Table S2.*** *Annual age-standardised hospitalisation rates (per 100,000 population) for thoracic vertebral fractures (ICD-10-GM S22.0), lumbar vertebral fractures (ICD-10-GM S32.0), and postmenopausal osteoporosis with osteoporotic fracture (ICD-10-GM M80.0) in Germany between 2010 and 2023. Age standardisation was performed using the German standard population 2022.*
