# Supplementary material for: Nationwide trends and demographic projections of hospitalisations for vertebral fractures and postmenopausal osteoporosis in Germany: a 24-year registry-based study
Source: Arch Osteoporos. 2026 Oct 30;21(1):154. doi: 10.1007/s11657-026-01796-5 (PMC13722723; doi:10.1007/s11657-026-01796-5)
Supplement: Supplementary file 3 [file 11657_2026_1796_MOESM3_ESM.docx]

**Nationwide Trends and Demographic Projections of Hospitalisations for Vertebral Fractures and Postmenopausal Osteoporosis in Germany: A 24-Year Registry-Based Study**

Houmam Anees^1,2^*****, Khaled Qadourah^1^, Goran Georgievski^1^, Christian Heiss^1,2,3^ and Thaqif El Khassawna^2,4^

^1^Department of Trauma, Hand and Reconstructive Surgery, Faculty of Medicine, Justus-Liebig-University of Giessen, 35392 Giessen, Germany

^2^Experimental Trauma Surgery, Faculty of Medicine, Justus-Liebig-University of Giessen, 35392 Giessen, Germany

^3^Biruni University, Istanbul, Türkiye

^4^School of Pharmacy, The University of Jordan, Amman 11942, Jordan

***Corresponding Author:** [Houmam.Anees@chiru.med.uni-giessen.de](mailto:Houmam.Anees@chiru.med.uni-giessen.de) (H. Anees).

| **Diagnosis** | **Age group** | **2000** | **2005** | **2010** | **2015** | **2020** | **2023** |
| --- | --- | --- | --- | --- | --- | --- | --- |
| S22.0 | <15 | 4 | 5 | 6 | 6 | 4 | 3 |
|  | 15–44 | 11 | 12 | 12 | 11 | 10 | 9 |
|  | 45–64 | 15 | 18 | 22 | 22 | 19 | 19 |
|  | ≥65 | 48 | 60 | 89 | 108 | 98 | 107 |
| S32.0 | <15 | 2 | 2 | 2 | 2 | 1 | 1 |
|  | 15–44 | 16 | 16 | 16 | 14 | 12 | 12 |
|  | 45–64 | 31 | 34 | 37 | 35 | 32 | 31 |
|  | ≥65 | 111 | 136 | 169 | 194 | 170 | 183 |
| M80.0 | <15 | – | – | – | – | – | – |
|  | 15–44 | 0 | 0 | 0 | 0 | 0 | 0 |
|  | 45–64 | 2 | 2 | 3 | 3 | 3 | 3 |
|  | ≥65 | 17 | 38 | 68 | 75 | 65 | 60 |

***Supplementary Table S3.*** *Age-specific hospitalisation rates for thoracic vertebral fractures (ICD-10-GM S22.0), lumbar vertebral fractures (ICD-10-GM S32.0), and postmenopausal osteoporosis with osteoporotic fracture (ICD-10-GM M80.0) in Germany at selected time points between 2000 and 2023. Rates are presented per 100,000 population within the respective age group. Data were obtained from the German Federal Health Monitoring System (GBE).*

*–, not applicable.*
